# The Effects of Time-Restricted Eating on Metabolism and Gut Microbiota: A Real-Life Study

**DOI:** 10.3390/nu14132569

**Published:** 2022-06-21

**Authors:** Ilario Ferrocino, Marianna Pellegrini, Chiara D’Eusebio, Ilaria Goitre, Valentina Ponzo, Maurizio Fadda, Rosalba Rosato, Giulio Mengozzi, Guglielmo Beccuti, Fabio Dario Merlo, Farnaz Rahimi, Isabella Comazzi, Luca Cocolin, Ezio Ghigo, Simona Bo

**Affiliations:** 1 Department of Agricultural, Forestry and Food Science, University of Torino, 10095 Torino, Italy; ilario.ferrocino@unito.it (I.F.); lucasimone.cocolin@unito.it (L.C.); 2 Department of Medical Sciences, University of Torino, 10126 Torino, Italy; marianna.pellegrini@unito.it (M.P.); chiara.deusebio@unito.it (C.D.); ilaria.goitre@unito.it (I.G.); valentina.ponzo@unito.it (V.P.); giulio.mengozzi@unito.it (G.M.); guglielmo.beccuti@unito.it (G.B.); isabella.comazzi@unito.it (I.C.); ezio.ghigo@unito.it (E.G.); 3 Dietetic Unit, Città della Salute e della Scienza Hospital, 10126 Torino, Italy; mfadda@cittadellasalute.to.it (M.F.); fdmerlo@gmail.com (F.D.M.); frahimi@ittadellasalute.to.it (F.R.); 4 Department of Psychology, University of Torino, 10126 Torino, Italy; rosalba.rosato@unito.it

**Keywords:** time-restricted eating, obesity, diet, gut microbiota

## Abstract

The metabolic benefits of time-restricted eating (TRE) in humans are statistically significant but not clinically relevant. Few data are available about the effects of TRE on the gut microbiota. We compared the effects of a TRE regimen (<12 h feeding; *n* = 25) with a time-unrestricted (TUE) regimen (>12 h feeding; *n* = 24), on the clinical and dietary variables and gut-microbiota composition in patients with obesity, who were subjected for 12 weeks to the same caloric restriction. Median weight loss was 4.0 kg and 2.2 kg in the TRE and TUE groups, respectively, with a between-group borderline difference (*p* = 0.049). No significant between-group difference was found in other dietary, anthropometric, or laboratory variables. There were no substantial between-group differences in alpha and beta diversity or gut-microbiota composition. The TRE group showed a significant increase in the frequency of *Lachnospiraceae*, *Parasutterella*, and *Romboutsia* at the study’s end. A TRE regimen induced small changes both in metabolic/dietary variables and in the gut-microbiota composition, with respect to the TUE. The microbial changes we have found were of uncertain clinical significance.

## 1. Introduction

Time-restricted eating (TRE), or time-restricted feeding (TRF) when referring to animals, is the restriction of daily-caloric intake to a window ranging from 4 to 12 h to optimize nutrient utilization and metabolism [1]. Animal studies have consistently shown a favorable effect by TRF on preventing or reversing chronic metabolic diseases, while human studies on TRE are conflicting [2,3,4,5]. Overall, a statistically significant reduction in fasting glucose and weight was reported in comparison with unrestricted regimens, even if the clinical significance of these differences was not meaningful [2,3,4,5].

Regimens with food intake restricted to the early part of the day (early TRE) appeared to be more effective and better aligned with the physiological circadian rhythms involved in metabolism regulation [5]. However, the level of evidence was low, owing to methodological bias, low samples, limited follow-up, heterogeneity in the time of the day of food restriction, and high variability in the dietary intakes of the studies. Adhering to very restrictive regimens (i.e., >16 h of fasting) may be difficult in real life, outside of clinical trials, for work, family, and social reasons. Furthermore, many protocols allowed food intake in the late afternoon/evening [3,6], when beta-cell function has been reported to be reduced with subsequent post-meal hyperglycemia and hyperlipidemia [7,8,9,10].

The gut microbiome, which is the host microbial community with distinct metabolic properties, shows circadian variations in its composition, and animal studies have shown that TRF contributes to these daily cyclic fluctuations [11]. Few human studies on small numbers of subjects are at present available on this topic, showing that TRE increased abundance of *Akkermansia muciniphila* and the *Bacteroides fragilis* group [12] and enhanced gut-microbial richness, with enrichment of *Prevotellaceae* and *Bacteroideaceae* [6,13,14], particularly increasing the abundance of *Butyricicoccus pullicaecorum* [15]. On the other hand, other authors failed to find differences between time-restricted and unrestricted regimens [16], and animal experiments in pediatric mice even found disturbed microbiota–host relationships and persistent gut-flora impairments after TRF [17,18].

The purpose of the present study was, therefore, to assess the effects of TRE on metabolic variables and gut-microbiota composition, when compared to time-unrestricted eating (TUE), with the same caloric and nutrient composition in outpatients with obesity, in real life. By doing so, patients were left free to choose to adhere or not adhere to TRE, according to their possibilities and preferences linked to work and/or family commitments.

## 2. Materials and Methods

### 2.1. Participants

All patients who attended their first visit at the Obesity Unit of the “Città della Salute e della Scienza” Hospital, starting from January 2021, were assessed for eligibility.

The inclusion criteria were age 18–75 years, BMI ≥ 30 kg/m^2^ and <45 kg/m^2^, self-reported light-physical activity (<2 h/week), and a usual feeding time > 12 h/day, with the last meal of the day beginning later than 8:00 p.m.

The exclusion criteria were overnight-shift working, secondary causes of obesity (e.g., hypothalamic diseases, endocrine diseases, use of obesogenic drugs), previous bariatric surgery, known psychiatric disorders diagnosed by a physician (including known eating disorders), any acute or chronic disease, condition, or drug potentially impacting weight loss, substance or alcohol abuse within the last 12 months, antibiotic/prebiotic/probiotic use in the previous 2 months, current or planned pregnancy/breastfeeding, and inability to provide a written informed consent for study participation.

### 2.2. Ethical Aspects

Participants gave their informed consent for study participation. The protocol was approved by the local Ethics Committee (approval number: 677/2018; approval date: 13 November 2018) and was conducted in accordance with the Declaration of Helsinki principles.

### 2.3. Design of the Study

An experimental design was used, with assignment based on participants’ preference. We proposed the two regimes (TRE and TUE) to all participants, allowing them to choose one of them according to their ability to anticipate the last meal of the day. In Italy, dinner is generally eaten from 8:00 p.m. onwards and many people stop working after 7:30 p.m. Many of our participants had family or work situations that did not allow them to eat dinner before 7:00 p.m., as required by the TRE protocol (see below).

Therefore, participants were allowed to choose the restricted or unrestricted regimen, and no randomization was applied in group assignment. The first consecutive 25 eligible patients who agreed to be included in the TRE, and the first consecutive 25 eligible patients who agreed to be included in the TUE groups, were enrolled.

### 2.4. Dietary Intervention

Our outpatient unit was part of the public healthcare system, with low costs to the patients. The weight-loss program included assessments by expert endocrinologists, registered dietitians, and psychologists [19]. Group sessions were abolished in 2020, during the SARS-CoV-2 pandemic, and all visits were carried out at an individual level [20]. Verbal and written dietary, exercise, and behavior recommendations were provided to all participants.

An individually prescribed diet, according to the Mediterranean-diet principles, was provided (45–55% carbohydrates, <10% sugars, 30% fats, <10% saturated fats, 15–25% proteins, 20–30 g fiber) with an energy restriction ranging from 500 to 1000 kcal, based on the individual caloric requirements and usual consumption. Moderate activity, such as brisk walks for at least 150 min/week, plus 30 min/week of exercise against resistance, was recommended. Verbal and written advice on practical lifestyle tips (i.e., portion control, identification of common dietary mistakes, how to identify high-sugar, high-fat, and high-calorie foods, how to choose high-fiber foods, how to reduce the intakes of salt and food/beverages with added sugar, options for dining out and healthy shopping, how to increase daily exercise and include physical movement into habitual activities) were provided to all participants.

Participants were asked to weigh foods to ensure both adherence to the given recommendations and accurate reporting of their caloric intake. In the TRE group, participants were allowed to feed in an interval of <12 h, and the last meal of the day had to be finished by 7:00 p.m. In the TUE group, participants received the same nutritional recommendations but were allowed to continue eating as usual (>12 h), with no restrictions on the time of the meals. Therefore, the only between-group difference was the restriction (or not) of the feeding period. The intervention lasted 12 weeks in both groups. The use of medications affecting weight or energy balance was not allowed during the study.

### 2.5. Monitoring

Patients from both groups received face-to-face visits by the same trained endocrinologists and registered dietitians at enrollment, after 4 weeks and after 12 weeks. During each visit, the compliance to the assigned dietary regimen and the possible occurrence of adverse events were assessed. Furthermore, patients from both groups were contacted by telephone after 8 weeks by a dietician, to check adherence and adverse events due to the intervention. Finally, participants had to report every day the time of the first and last meal of the day, in order to assess the adherence to the meal-timing schedule.

The records were reviewed by the dieticians to verify the consistency with the feeding windows for each group; a participant was considered non-compliant if >3 days of non-adherence to the meal-timing schedule were reported during the 12-week period of the study.

Dietary habits were evaluated by a 3-day food record. All participants completed a detailed written food diary at the baseline and at the end of the study, recording everything they ate or drank during 2 consecutive weekdays and 1 weekend day. The 3-day food record data were loaded into Win Food Pro 3 software (Medimatica, Colonnella, Teramo, Italy), and the mean nutritional values for the 3 days were reported [8]. Due to the nature of the intervention and monitoring, neither participants nor researchers were blinded to the group assignment. However, blood sample and microbiota analyses were performed by laboratory personnel blinded to the group assignment.

### 2.6. Measurements

Weight and height were measured with the participants wearing light clothes and no shoes, to the nearest 0.1 kg and 0.1 cm, respectively, by a mechanical-column scale (SECA model 711, Hamburg, Germany) and a Stadiometer (SECA 220 measuring rod, Hamburg, Germany); waist and neck circumferences were assessed by a plastic-tape measure at the umbilicus level or under the cricoid cartilage, respectively. Arterial blood pressure (BP) was measured from the left arm, in a sitting position, after at least 10 min of rest, with a mercury sphygmomanometer with appropriate cuff sizes (ERKA Perfect-Aneroid, Germany). Two measurements were taken by trained personnel; the values reported were the means of the two measurements. Fat mass (FM) and fat-free mass (FFM) were determined by single- frequency bioelectrical impedance, in accordance with the equations of the manufacturer (BIA 101, Akern, Florence, Italy).

Indirect calorimetry (Q-NRG Metabolic Monitor, Cosmed, Rome, Italy) was used to assess resting metabolic rate (RMR), by measuring the inspired and expired concentrations of oxygen (VO_2_) and carbon dioxide (VCO_2_), which reflect nutrient metabolism. The canopy was placed over the face and carefully checked to prevent air leakage. The exams were performed in a quiet room with a temperature kept at 23–25 °C, with patients in a supine position, who had been fasting for at least 12 h and were awake, motionless, and silent. The respiratory quotient was the ratio between VCO_2_ and VO_2_ (VCO_2_/VO_2_). Blood samples were collected after an overnight fast. Laboratory measurements were centralized and have been previously described [21].

Stool samples were self-collected by the patients as previously described [22]. Briefly, participants were instructed to self-collect the samples, and all materials were provided in a convenient, refrigerated, specimen-collection kit. Patients were provided with sterile containers to collect the feces (VWR, Milan, Italy). Fecal samples were collected at home and transferred into the sterile sampling containers using a polypropylene spoon (~10 g) and immediately stored at 4 °C. The specimens were transported to the laboratory within 8 h of collection under refrigerated temperature. Containers were immediately stored at −80 °C for DNA extraction. No storage medium was used.

### 2.7. DNA Extraction, Meta-Taxonomic Amplicon Sequencing, and Bioinformatic Analyses

DNA extraction from 1000 mg of fecal samples was carried out following the SOP 07 guidelines and procedure developed by the International Human Microbiome Standard Consortium (http://www.microbiome-standards.org/index.php?id=Sop&num=007, accessed on 1 February 2021). RNAse treatment was then performed on the extracted DNA, quantified by using the QUBIT dsHS kit, and standardized at 5 ng/μL.

The V3-V4 region of the 16S rRNA was amplified using the primers 16SF (5′-TCGTCGGCAGCGTCAGATGTGTATAAGAGACAG-3′) and 16SR (5′-GTCTCGTGGGCTCGGAGATGTGTATAAGAGACAG-3′) [23], according to the Illumina 16S Metagenomic Sequencing Library Preparation instructions.

Amplicons were then purified, tagged, normalized, and pooled according to the Illumina protocols. DNA libraries were sequenced on the Illumina MiSeq platform, leading to 2 × 250 bp paired-end reads. After sequencing, raw reads were imported in QIIME2 software (https://docs.qiime2.org/, accessed on 4 April 2022) for quality, with the chimera-filtering step by the qiime dada2 denoise-paired script [24]. The amplicon-sequence variants (ASVs) obtained were then used for taxonomic assignment against the SILVA database. QIIME2-diversity script was then used in order to calculate alpha and beta diversity.

### 2.8. Statistical Analyses

Based on the literature data [25], we hypothesized to observe a mean percent difference in weight (end of the study minus the baseline) of 10% in the TRE group compared to the TUE group. Assuming a common standard-deviation value of 0.12, alpha = 0.05, and a power of 0.80, 24 participants per group are needed. The number was rounded to 25 per group, based on the dropouts of our previous intervention trials (0% after 12 months [26] and 0% after ~12 weeks [27]).

The Kolmogorov–Smirnov test was used to test normality of the data. Within-group and between-groups differences for metabolic variables were assessed respectively by *t*-test for paired data (or Wilcoxon matched-pairs test) or by t-Student’s test (or Mann–Whitney test), as appropriate. We calculated changes in the variables (deltas) as: final value minus baseline value of the variables.

Most delta values were not-normally distributed. In order to be more conservative (considering that often in one group the delta values were non-normally distributed and, in the comparison group, the delta values were normally distributed), we used a non-parametric test for all the comparisons of delta values.

The Bray–Curtis distance matrix was used to perform the pairwise PERMANOVA analysis by groups. The ASV table was imported in the R environment to perform pairwise comparisons by using the Wilcoxon rank-sum test. ASVs with a frequency of 100 in at least 20 samples were considered only. Venn diagrams were obtained by Venn Diagram Maker (https://goodcalculators.com/venn-diagram-maker/, accessed on 26 April 2022).

Spearman’s correlations were used to study the relationships between ASVs and metabolic variables, using the psych package, and plotted through the function corrplot of R. Multiple-regression analyses were performed to evaluate the associations between bacterial ASVs as well as nutrient and clinical variables after the dietary interventions, after adjusting for age, gender, and the value of the variable at enrollment (Statistica, ver. 7.0; StatSoft Inc., Tulsa, OK, USA).

## 3. Results

The flow of the study is described in Figure S1. One patient from the TUE dropped-out for personal reasons. The baseline characteristics of participants are reported in Table 1. Participants were mostly middle-aged women; patients in the TRE group were slightly older than those in the TUE group, although without statistical significance (Table 1). No significant difference was evident between groups for dietary intakes or anthropometric and clinical variables (Table 1).

All participants from the TRE group reported to be compliant with the fasting hours required by the protocol and to have finished dinner by 7:00 p.m. Median feeding hours were 11 and 13, respectively, in the TRE and TUE groups (*p* < 0.0001). No adverse event was reported by a participant from either group.

### 3.1. Changes in Metabolic Variables

After a 12-week intervention, a significant within-group reduction in weight, BMI, fat mass, waist and neck circumference, systolic blood pressure, serum-fasting glucose and alanine-aminotransferase levels was observed in both groups (Table 2).

Patients reduced their caloric intake by ~500 kcal, with an overall improvement in their dietary intakes: proteins (% total calories) increased and saturated fats (% total calories) decreased within each group (Table 2). TRE participants only showed an increased intake of polyunsaturated fats (% total calories). Median weight loss was 4.0 kg and 2.2 kg in the TRE and TUE groups, respectively, with a borderline between-group difference. The median percentage of weight loss of −4.7% and −2.3%, respectively (*p* = 0.038). Between-group differences in other anthropometric, dietary, and laboratory variables (delta) were not observed (Table 2).

### 3.2. Microbiota Analysis

**Figure 1:**
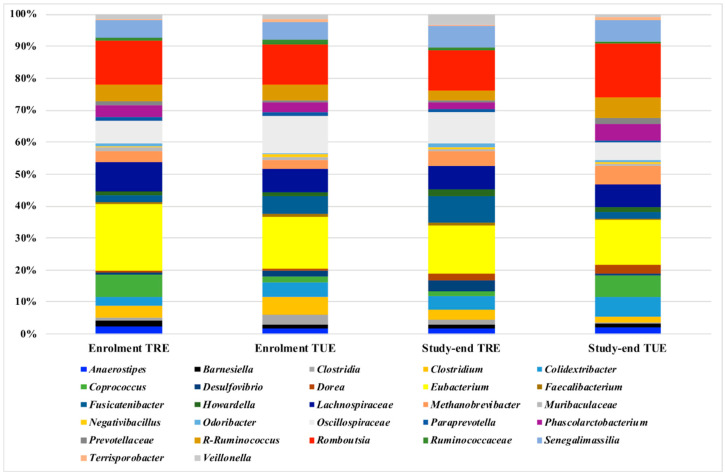
Microbiota composition detected by 16S amplicon-target sequencing. Only ASVs with a frequency of 100 in at least 20 samples are shown. Frequency of ASVs in the 4 study groups (TRE enrollment, TRE study end, TUE enrollment, TUE study end) were shown.

After sequencing and denoising, a total of 1,286,232 reads were obtained with an average 12,862 reads/sample and a sample coverage > 99%. Alpha and beta diversity results were not significantly different between the TRE and TUE groups (data not shown). The Bray–Curtis distance matrix showed no clear separation of the samples according to the dietary regimen (PERMANOVA pairwise *p* > 0.05). The microbiota composition at the lowest taxonomic level (Figure 1) showed the predominance of *Eubacterium*, *Romboutsia*, *Lachnospiraceae*, *Oscillospiraceae*, *Coprococcus*, *R-Ruminococcus*, and *Methanobrevibacter* in all the analyzed samples with no significant differences according to the dietary regimen.

**Figure 2:**
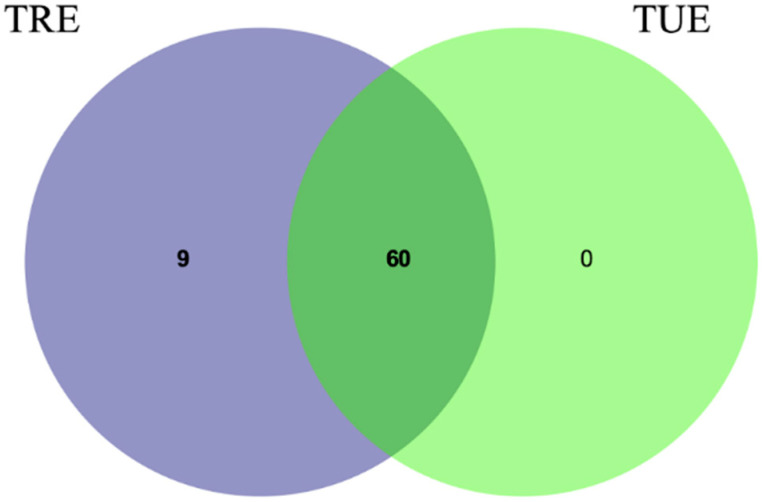
Venn diagram shows overlap of bacterial ASVs at the study end in TRE and TUE groups.

ASVs were analyzed by a Venn diagram analysis in order to detect unique ASVs or shared ones. Within-group comparisons did not allow to detect any unique ASVs (Figure 2). Between-group comparisons showed no significant differences at the baseline, but, at the end of the study, the unique presence of *Anaerovoracaceae*, *Christensenellaceae*, *Lactobacillus*, *Megasphaera*, *Lachnospira*, *Turicibacter*, *Butyricicoccus*, *Catenibacterium* and *Escherichia* were detected in the TRE cohort.

**Figure 3:**
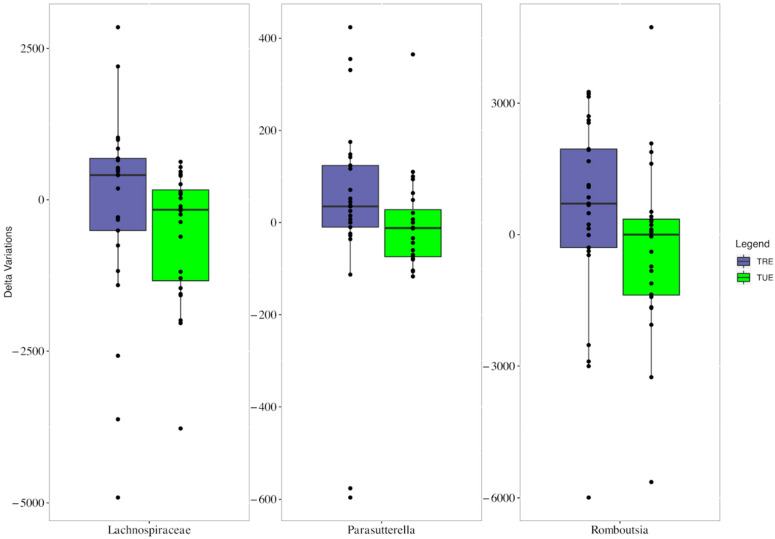
Boxplots showing the mean changes (study end values minus enrollment values) of Lachnospiraceae, Parasutterella, and Romboutsia in TRE (very pery bars) and TUE groups (green bars).

Median changes in *Lachnospiraceae*, *Parasutterella*, and *Romboutsia* were significantly different between groups, with increased frequencies in the TRE group (Figure 3).

### 3.3. Correlations between Microbiota as well as Metabolic and Dietary Variables

Many significant correlations were found between ASVs as well as metabolic and dietary variables at the end of the study in both the TRE and TUE groups, by Spearman’s correlations (Figure S2). In a multiple-regression model, after adjustments for age, gender, and the variable value at enrollment, a few significant associations were found in each group (Table S1). In particular, negative associations between *Fusicatenibacter* and diastolic blood pressure, *Lachnospiraceae* and triglycerides, and *Oscillospiraceae* and glycated hemoglobin were evident in the TRE group. In the TUE group, an inverse association between *Paraprevotella* and serum triglycerides was detected.

## 4. Discussion

A time-restricted eating regimen was barely more effective than the time-unrestricted eating regimen, with regard to weight loss, while no significant differences in other anthropometric, metabolic, and dietary variables were detected. Furthermore, small differences in the microbiota composition were found between the time-restricted and unrestricted regimens.

### 4.1. TRE and Metabolic Changes

The human literature on the metabolic effects of TRE is conflicting, since the benefits on weight and fasting glucose reductions were statistically significant, but not clinically significant [2,3,4,5]. Overall, shorter and smaller studies found beneficial effects on weight loss and the metabolic pattern after TRE [28], while larger trials with longer follow-up failed to find relevant differences [29,30]. Furthermore, adverse metabolic effects have been reported with TRE by a few studies [31,32,33]. Our data were very similar to the findings of recent, large, and randomized controlled trials. In particular, Liu found a non-significant between-group difference of 1.8 kg of weight and 1.3% fat mass between time-restricted and unrestricted regimens in Chinese patients with obesity, after 12 months of follow-up [29]. Similarly, Lowe reported non-significant between-group differences in weight (0.26 kg) and whole-body fat mass (−0.48 kg) in a US cohort after 12 weeks of follow-up [30].

Our data were very similar to the findings of recent, large, randomized controlled trials, showing a <2 kg difference in weight loss and around a 1% difference in percentage of fat-mass reduction between the time-restricted and unrestricted regimens, in patients with obesity [29,30]. It should be noted that our two groups, although not randomized, were extremely similar at the baseline for anthropometric, dietary, and clinical variables and received the same lifestyle recommendations, with analogue changes in dietary habits being reported during the study (i.e., reduced energy intakes of ~500 kcal, increased protein consumption, decreased saturated fat consumption) (Table 2). Furthermore, energy expenditure did not significantly change between groups, in line with previous studies and metanalysis [5,30].

The lack of meaningful between-group differences supports the overwhelming benefit of calorie restriction over the effects of meal timing, contrarily to the hypothesis of the beneficial, intrinsic metabolic effects of TRE, based on the realignment of the circadian clock and improvements in insulin sensitivity and metabolic pathways [28,34,35,36]. Accordingly, most of the pathways known to mediate the benefits of fasting are activated more by caloric restriction than by time restriction [35], and the possibility that TRE benefits may be confounded by reduced-energy intake is still a matter of debate [37].

Human studies reported heterogeneous feeding-time intervals ranging from 4 to 12 h, with preferential food consumption either in the morning–early afternoon or afternoon–early evening or even exclusively in the evening [1,2,3,4,5,28]. The efficacy of TRE seems to depend on the time of the eating window, with greater benefits for shorter windows [35] or earlier regimens [5]. Our feeding window (median 11 h) was wider than those reported in the literature (6–10 h), with a small difference between the two groups (11 vs. 13 h), and this might have reduced the benefits of TRE.

Indeed, this is a real-life study and, in Italy, reaching tighter and earlier feeding intervals concurrent with the active phase of the day was very difficult, since the last meal of the day is usually eaten from 8:00 p.m. onwards. Furthermore, a 10 h eating window has been shown to provide comparable health benefits to shorter windows of 6–8 h, without the mild adverse effects associated with prolonged fasting regimens [35]. Therefore, the feeding interval of our TRE group did not differ significantly from the one that has proven to be effective, and the TRE group was allowed to obtain an optimal self-reported adherence to the TRE regimen under free-living conditions, without any adverse effects. Most rigorous trials with controlled-feeding protocols required supervision and strict monitoring as well as imposed dietary regimens very different from usual lifestyle habits [38]. This, in turn, made the recruitment challenging and resulted in the inclusion of very selected groups of participants [38].

It could be hypothesized that we did not obtain striking results because our time window was later than in other studies, in which the last meal occurred in the first half of the afternoon [5]. Indeed, recent studies on early TRE did not find meaningful effects of this regimen as well [28,39]. The increased cardiometabolic risk of people with circadian misalignment (i.e., shift workers, individuals who often eat at night) is well known [40,41]. TRE was hypothesized to address circadian-rhythm disruption and the related metabolic impairments [35]. The beneficial effects of time-restricted feeding in animals, indeed, seem to be less evident in humans. There is great heterogeneity among the studies in the proposed dietary regimens, both in the timing as well as the caloric and macronutrient composition, ranging from ad libitum intakes to very-low-caloric diets, and this could have sustained the discrepancies found in the literature.

Furthermore, the possibility that different human chronotypes could respond differently to time-restricted regimens should be considered. Indeed, it has been recently demonstrated that the beginning of the biological night may largely differ depending on the chronotype [10]. “Early” chronotypes display early melatonin (hormone with a central role in the circadian system and in the biological night) onset, i.e., around 7:00 p.m.; “late” chronotypes have melatonin onset much later (even at 1:00 a.m.) [10]. Therefore, dinner at 7:00 p.m. is an early-circadian dinner for the latter group, but not for the early chronotypes, and the potential benefits of a TRE regimen might be more evident when the external timing (clock timing) is synchronized with the internal timing, i.e., the specific individual-circadian pattern.

### 4.2. TRE and Microbiota Changes

Owing to the influences of the gut microbiota on energy homeostasis, nutrients absorption, appetite regulation, metabolic and inflammatory pathways, and circadian alignment, it was conceivable that time-restricted regimes could influence its composition. It is well-known that the human-gut microbiota shows circadian rhythmicity in its composition; TRE might have an impact on the daily, cyclic microbiome fluctuations, to restore the rhythm perturbation induced by unhealthy lifestyle habits [1,6,11].

A few human studies are available with contrasting results [6,12,13,14,15,16]. We failed to find between-group differences in gut-microbial alpha diversity, contrarily to authors showing enhanced values with TRE [6] but in line with others [15,16]. We cannot rule out that our fasting interval could have played a role, since an early TRE (with the last meal of the day at 3:00 p.m.) determined a significant increase in alpha diversity, while a later TRE (with the last meal at 8:00 p.m.) failed to demonstrate any differences in bacterial richness versus the control group’s unrestricted eating [14]. However, a recent observational study contradicted this hypothesis by demonstrating that Ramadan fasting, with restricted feeding from dawn to sunset, significantly increased bacterial richness [42].

At the end of the study, we found few ASVs that were unique to the TRE group, which, however, included both bacteria producing short-chain fatty acids (such as *Butyricicoccus*, *Lactobacillus*, and *Lachnospira*) and bacteria with less-favorable effects (such as *Christensenellaceae* and *Escherichia*). Furthermore, in the TRE group, we detected statistically significant increases in ASVs, such as *Romboutsia*, *Parasutterella*, and *Lachnospiraceae*, which appear to have contrasting effects on human metabolism. Indeed, *Romboutsia*, *Parasutterella* and *Lachnospiraceae* have been reported to be associated either directly (with a predisposing effect) [43,44,45,46,47] or inversely (with a protective effect) [43,48,49,50,51] with metabolic abnormalities.

We found no significant associations between these ASVs and dietary or anthropometric variables, suggesting that the changes in the ASVs were not related to weight or dietary variations but to changes in timing of meals. The relevance of the changes in the gut microbiota we have detected is hard to define at present, since the impact on human health of the implicated genera is still not clarified. The upregulation of *Lachnospiraceae* has already been described after Ramadan and interpreted as an obvious possible mechanistic explanation for the health benefits of TRE, given their butyric-acid-producing capacity [42]. Other authors have reported either an increased abundance of butyrate-producing bacteria [13] or no relevant difference in the relative abundances of such taxa [14,16] after TRE regimens. In particular, either an enrichment [6] or a decreased abundance [42] in Prevotellaceae have been described.

Again, differences in timing, composition of meals, and quantity and quality of nutrients might have determined these divergent results. Animal studies are highly contrasting too. An increased abundance of several microbial enzymes involved in carbohydrate and protein metabolism, expressed by *Lactobacillus* and *Akkermansia muciniphila*, have been reported in rat-fecal microbiota after TRF [52], and this regimen was able to counteract the detrimental effects of a high-fat diet by microbiota modulation and restoration of the circadian rhythm of the gut-microbiota composition [53]. On the other hand, disturbed microbiota–host relationships and persistent gut-flora impairments after TRF were described in pediatric mice [17,18].

Owing to the small number of studied patients, the medium/high risk of bias of the available studies, and the current low level of evidence [54], further studies, assessing the mechanistic and functional aspects of microbiota–host relationships, are needed to better define the impact of TRE on human-gut microbiota.

### 4.3. Limitations and Strengths

The low number of participants, the short follow-up, and the lack of systems of lifestyle monitoring, such as the employment of telephone applications or devices to assess physical activity, were all limitations of the present study. Self-reported energy intake is often inaccurate; however, the reported intakes were compatible with the objective measurements of RMR. Finally, the study was not randomized; indeed, the two groups resulted in being well-matched for all the variables at the baseline, and no significant between-group difference was found.

Our strengths were the low drop-out rate, the data completeness, the fact that the participants were free-living, which increases the external validity of the study, and the concomitant evaluation of energy expenditure and microbiota composition.

## 5. Conclusions

A time-restricted eating regimen induced small changes both in metabolic/dietary variables and in the gut-microbiota composition, with respect to a nutritional regimen that was comparable in nutrient and caloric composition but was not time-restricted. It could be useful to test whether the stratification of patients by chronotype could impact the results, since a tailored fasting window might be more appropriate for the specific chronotype of each individual.

## Figures and Tables

**Table 1:** Baseline characteristics by dietary regimen.

	All	TRE	TUE	*p*
Number	49	25	24	
Age (years)	56.9 ± 8.0	58.6 ± 7.9	55.1 ± 7.9	0.13
Males (%)	18.4	20.0	16.7	0.76
Height (m)	1.61 ± 0.08	1.60 ± 0.09	1.61 ± 0.08	0.72
Weight (kg)	91.3 ± 12.7	90.4 ± 11.8	92.2 ± 13.7	0.62
BMI (kg/m^2^)	35.3 ± 3.1	35.2 ± 3.0	35.4 ± 3.2	0.78
Waist circumference (cm)	114.9 ± 10.6	117.1 ± 9.5	112.7 ± 11.4	0.15
Neck circumference (cm)	38.6 ± 3.4	39.3 ± 3.2	37.9 ± 3.4	0.14
Body-fat mass (kg)	37.6 ± 8.3	37.1 ± 8.9	38.1 ± 7.7	0.69
Body-lean mass (kg)	53.2 ± 10.7	53.1 ± 12.4	53.3 ± 9.0	0.95
Fat mass (%)	41.3 ± 7.9	41.3 ± 9.7	41.2 ± 5.6	0.98
Systolic blood pressure (mmHg)	134.5 ± 13.4	135.4 ± 11.7	133.5 ± 15.2	0.63
Diastolic blood pressure (mmHg)	82.7 ± 7.6	82.8 ± 5.4	82.5 ± 9.4	0.89
Calorimetric data				
RMR (kcal)	1474.6 ± 269.0	1469.8 ± 265.4	1479.5 ± 278.3	0.90
Respiratory quotient	0.76 (0.06)	0.76 (0.07)	0.76 (0.08)	0.36 *
Dietary intakes				
Total energy (kcal/day)	2086.6 ± 399.7	2086.8 ± 429.7	2086.5 ± 375.3	0.99
Carbohydrates (% total kcal)	51.2 ± 6.2	49.9 ± 5.8	52.5 ± 6.4	0.14
Proteins (% total kcal)	14.0 ± 1.6	13.9 ± 1.8	14.1 ± 1.5	0.77
Fats (% total kcal)	31.7 ± 6.1	32.9 ± 5.1	30.5 ± 6.9	0.18
Saturated fats (% total kcal)	8.4 ± 1.7	8.7 ± 1.7	8.1 ± 1.6	0.16
Monounsaturated fats (% total kcal)	17.4 ± 3.9	18.1 ± 3.2	16.8 ± 4.5	0.24
Polyunsaturated fats (% total kcal)	4.7 ± 1.3	4.7 ± 1.3	4.6 ± 1.3	0.71
Fiber (g/day)	28.3 ± 5.9	27.3 ± 6.3	29.2 ± 5.4	0.27
Laboratory variables				
Fasting glucose (mg/dL)	102.5 ± 22.5	102.6 ± 18.2	102.5 ± 26.7	0.98
Glycated hemoglobin (mmol/mol)	40.4 ± 6.0	40.4 ± 4.3	40.5 ± 7.5	0.96
Total cholesterol (mg/dL)	204.7 ± 39.9	204.5 ± 39.5	204.9 ± 41.1	0.97
HDL cholesterol (mg/dL)	52.0 ± 12.2	51.2 ± 10.0	52.9 ± 14.4	0.62
Triglycerides (mg/dL)	133.3 ± 56.1	141.3 ± 54.3	125.0 ± 58.0	0.32
Alanine aminotransferase (IU/L)	27.0 (16.0)	26.0 (30.0)	28.5 (10.5)	0.58 *

Mean ± SD for normally distributed variables; median (interquartile range) for variables not normally distributed; * Mann–Whitney test.

**Table 2:** Changes in the variables at 12 weeks by dietary regimen.

	TRE		TUE		
	After	Median Delta	After	Median Delta	*p* ^†^
Number	25		24		
Weight (kg)	86.0 ± 11.7 **	−4.00	89.2 ± 13.6 **	−2.20	0.049
BMI (kg/m^2^)	33.5 ± 3.3 **	−1.60	34.1 ± 3.1 **	−0.86	0.096
Waist circumference (cm)	110.2 ± 7.8 **	−5.00	107.0 ± 8.8 **	−4.50	0.670
Neck circumference (cm)	37.5 ± 2.9 **	−1.50	36.5 ± 3.2 **	−1.00	0.332
Body fat mass (kg)	33.6 ± 8.0 **	−2.80	36.0 ± 7.7 *	−1.75	0.280
Body lean mass (kg)	52.2 ± 9.4	−0.90	52.9 ± 8.8	−0.30	0.490
Fat mass (%)	39.0 ± 7.5 *	−1.29	40.2 ± 5.3	−0.61	0.447
Systolic blood pressure (mmHg)	126.8 ± 11.3 **	−10.0	126.5 ± 18.1 **	−2.05	0.430
Diastolic blood pressure (mmHg)	82.2 ± 6.9	0.00	80.8 ± 11.5	0.00	0.516
Calorimetric data					
RMR (kcal)	1493.8 ± 242.6	+15.0	1480.2 ± 317.8	+22.0	0.936
Respiratory quotient	0.77 (0.05)	0.00	0.77 (0.08)	+0.02	0.306
Dietary intakes					
Total energy (kcal/day)	1521.5 ± 198.7	−497.3	1564.6 ± 259.7	−527.9	0.729
Carbohydrates (% total kcal)	50.9 ± 5.0	+1.33	52.5 ± 5.6	−0.54	0.631
Proteins (% total kcal)	15.5 ± 1.7 **	+1.40	15.6 ± 1.9 **	+1.59	0.984
Fats (% total kcal)	32.2 ± 4.5	−1.08	30.3 ± 4.6	−0.23	0.689
Saturated fats (% total kcal)	7.7 ± 1.5 **	−0.50	7.1 ± 1.0 *	−0.30	0.317
Monounsaturated fats (% total kcal)	17.8 ± 2.6	−0.29	16.8 ± 3.0	−0.34	0.779
Polyunsaturated fats (% total kcal)	5.3 ± 1.3 *	+0.57	5.0 ± 1.1	+0.26	0.575
Fiber (g/day)	29.1 ± 4.7	+1.13	30.3 ± 4.1	+0.93	0.984
Laboratory variables					
Fasting glucose (mg/dL)	85.0 ± 15.3 **	−19.0	84.3 ± 30.0 **	−10.5	0.764
Glycated hemoglobin (mmol/mol)	39.5 ± 4.6	−1.00	39.2 ± 7.9	−0.45	0.617
Total cholesterol (mg/dL)	191.7 ± 46.0	0.00	195.3 ± 43.7	−3.50	0.412
HDL cholesterol (mg/dL)	53.8 ± 11.6	+3.00	54.6 ± 12.3	+1.00	0.225
Triglycerides (mg/dL)	120.4 ± 46.5	−20.0	110.8 ± 51.8	−7.50	0.589
Alanine aminotransferase (IU/L)	24.0 (11.0) ^$^	−2.00	22.5 (8.0) ^$^	−5.00	0.992

Mean ± SD for normally distributed variables; median (interquartile range) for variables not normally distributed; * *t*-test for paired data: *p* < 0.05; ** t for paired data: *p* < 0.01; ^$^ Wilcoxon matched pairs test < 0.05; ^†^ differences between deltas were assessed by Mann–Whitney test.

## Data Availability

The data generated by sequencing were deposited in the National Center for Biotechnology Information (NCBI) Sequence Read Archive (SRA) and are available under the BioProject Accession Number PRJNA841785.

## Supplementary Materials

The following supporting information can be downloaded at: https://www.mdpi.com/article/10.3390/nu14132569/s1. Figure S1: Flow chart of the study; Figure S2: Spearman’s correlation between amplicon sequence variants (ASVs) as well as metabolic and dietary variables at the end of the study, in TRE (plot A) and TUE (plot B) groups. ASVs with a frequency of 100 in at least 20 samples and only significant associations are shown. The intensity of the colors represents the degree of correlation, where the blue color represents a positive degree of correlation and the red color a negative correlation. Supplementary Table S1: Statistically significant associations between microbiota composition as well as dietary and metabolic variables at the end of the study, in groups.
