# Supplementary material for: The Effects of Time-Restricted Eating on Metabolism and Gut Microbiota: A Real-Life Study
Source: Nutrients. 2022 Jun 21;14(13):2569. doi: 10.3390/nu14132569 (PMC9267969; doi:10.3390/nu14132569)
Supplement: Supplementary file 1 [file nutrients-14-02569-s001.zip › Supplementary Table S1.pdf]

Supplementary Table S1. Statistically significant associations between microbiota composition and dietary and metabolic variables at the study end by groups

	Rho	Beta	95%CI	p
**TRE**				
Fats (% total energy)*				
Dorea	0.44	15.9	1.78 30.0	0.039
Diastolic blood pressure**				
Fusicatenibacter	-0.43	-0.015	-0.025 -0.005	0.010
Triglycerides**				
Lachnospiraceae	-0.48	-0.025	-0.044 -0.006	0.022
Glycated hemoglobin**				
Oscillospiraceae	-0.42	-0.0015	-0.0027 -0.0003	0.022
**TUE**				
Triglycerides**				
Paraprevotella	-0.56	-0.121	-0.199 -0.043	0.007

Spearman's correlations (left) and multiple regression analyses (right)
*Multiple regression model evaluating the association between bacteria (dependent variables) and the specific nutrient (independent variable), after adjusting for age and gender
**Multiple regression model evaluating the association between metabolic variables (dependent variables) and bacteria (independent variables) after adjusting for age, gender, and the value of the variable at baseline. Each row is a model.
