# Supplementary figures and images for: The Effects of Time-Restricted Eating on Metabolism and Gut Microbiota: A Real-Life Study
Source: Nutrients. 2022 Jun 21;14(13):2569. doi: 10.3390/nu14132569 (PMC9267969; doi:10.3390/nu14132569)

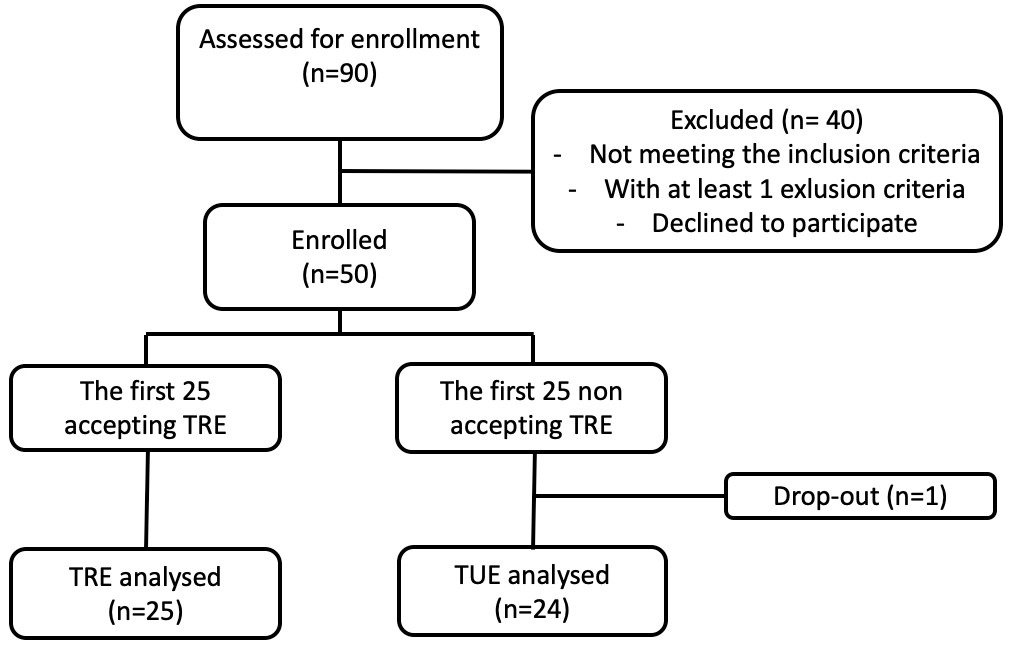

Supplement: Supplementary file 1 [file nutrients-14-02569-s001.zip › Figure S1.tif]

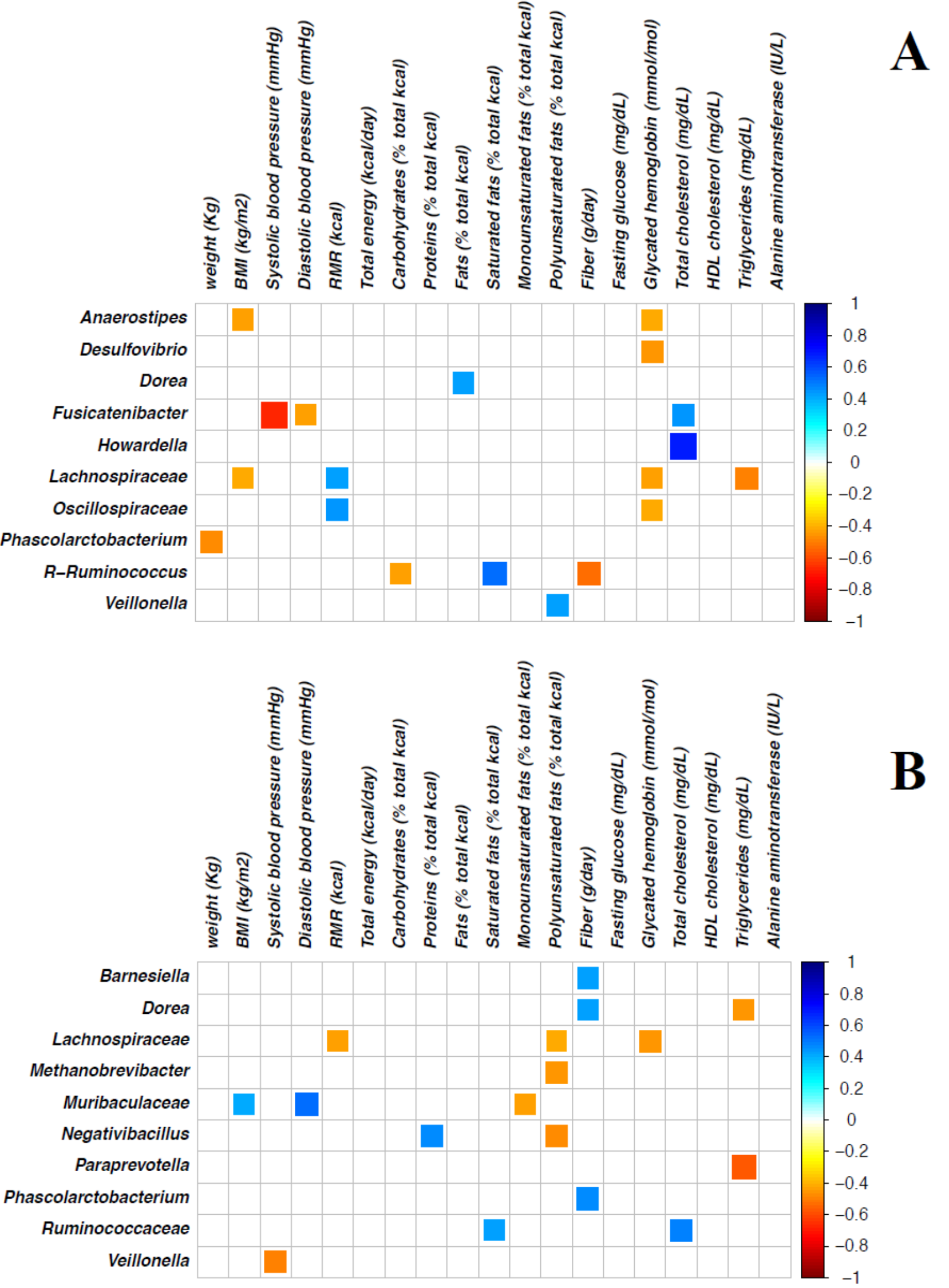

Supplement: Supplementary file 1 [file nutrients-14-02569-s001.zip › Figure S2.tif]
